# Compartmentation of photosynthesis gene expression in C_4_ maize depends on time of day

**DOI:** 10.1093/plphys/kiad447

**Published:** 2023-08-09

**Authors:** Ana Rita Borba, Ivan Reyna-Llorens, Patrick J Dickinson, Gareth Steed, Paulo Gouveia, Alicja M Górska, Celia Gomes, Johannes Kromdijk, Alex A R Webb, Nelson J M Saibo, Julian M Hibberd

**Affiliations:** Department of Plant Sciences, University of Cambridge, Cambridge CB2 3EA, UK; Instituto de Tecnologia Química e Biológica António Xavier, Universidade Nova de Lisboa, Oeiras 2780-157, Portugal; Instituto de Biologia Experimental e Tecnológica, Oeiras 2780-157, Portugal; Department of Plant Sciences, University of Cambridge, Cambridge CB2 3EA, UK; Department of Plant Sciences, University of Cambridge, Cambridge CB2 3EA, UK; Department of Plant Sciences, University of Cambridge, Cambridge CB2 3EA, UK; Instituto de Tecnologia Química e Biológica António Xavier, Universidade Nova de Lisboa, Oeiras 2780-157, Portugal; Instituto de Biologia Experimental e Tecnológica, Oeiras 2780-157, Portugal; Instituto de Tecnologia Química e Biológica António Xavier, Universidade Nova de Lisboa, Oeiras 2780-157, Portugal; Instituto de Biologia Experimental e Tecnológica, Oeiras 2780-157, Portugal; Instituto de Tecnologia Química e Biológica António Xavier, Universidade Nova de Lisboa, Oeiras 2780-157, Portugal; Instituto de Biologia Experimental e Tecnológica, Oeiras 2780-157, Portugal; Department of Plant Sciences, University of Cambridge, Cambridge CB2 3EA, UK; Department of Plant Sciences, University of Cambridge, Cambridge CB2 3EA, UK; Instituto de Tecnologia Química e Biológica António Xavier, Universidade Nova de Lisboa, Oeiras 2780-157, Portugal; Instituto de Biologia Experimental e Tecnológica, Oeiras 2780-157, Portugal; Department of Plant Sciences, University of Cambridge, Cambridge CB2 3EA, UK

## Abstract

Compared with the ancestral C_3_ state, C_4_ photosynthesis occurs at higher rates with improved water and nitrogen use efficiencies. In both C_3_ and C_4_ plants, rates of photosynthesis increase with light intensity and are maximal around midday. We determined that in the absence of light or temperature fluctuations, photosynthesis in maize (*Zea mays*) peaks in the middle of the subjective photoperiod. To investigate the molecular processes associated with these temporal changes, we performed RNA sequencing of maize mesophyll and bundle sheath strands over a 24-h time course. Preferential expression of C_4_ cycle genes in these cell types was strongest between 6 and 10 h after dawn when rates of photosynthesis were highest. For the bundle sheath, DNA motif enrichment and gene coexpression analyses suggested members of the DNA binding with one finger (DOF) and MADS (MINICHROMOSOME MAINTENANCE FACTOR 1/AGAMOUS/DEFICIENS/Serum Response Factor)-domain transcription factor families mediate diurnal fluctuations in C_4_ gene expression, while *trans*-activation assays in planta confirmed their ability to activate promoter fragments from bundle sheath expressed genes. The work thus identifies transcriptional regulators and peaks in cell-specific C_4_ gene expression coincident with maximum rates of photosynthesis in the maize leaf at midday.

## Teaser

The spatial partitioning of C_4_ gene expression between mesophyll and bundle sheath strands is dependent on time of day.

## Introduction

In hot and dry environments, C_4_ species can maintain higher rates of photosynthesis and operate higher water and nitrogen use efficiencies than plants that use the ancestral C_3_ cycle (Ghannoum et al. 2010). In C_3_ species, the inability of Rubisco to completely distinguish between carbon dioxide (CO_2_) and oxygen (O_2_) leads to competing carboxylation and oxygenation reactions. As temperatures increase and water availability is reduced, the oxygenation activity of Rubisco becomes more prevalent and so compromises photosynthetic efficiency (Lorimer 1981; Sedelnikova et al. 2018). More than 60 lineages of land plants have convergently evolved C_4_ photosynthesis, and despite some variation in how they concentrate CO_2_ in the leaf, in all cases, the likelihood of O_2_ reacting with Rubisco at the active site of the enzyme is reduced and carbon and energy losses associated with photorespiration suppressed (Bowes et al. 1971; Hatch 1987; Sage 2004).

Most C_4_ leaves possess Kranz anatomy, which consists of extensive vascularization combined with an inner wreath of bundle sheath cells and an outer ring of mesophyll cells (Haberlandt 1904; Langdale 2011). In C_4_ plants with this leaf anatomy, photosynthetic reactions are normally partitioned between mesophyll and bundle sheath cells. Atmospheric CO_2_ is first converted to bicarbonate (HCO_3_^−^) by carbonic anhydrase (CA) and then assimilated into a 4-carbon acid by the O_2_-insensitive phosphoenolpyruvate carboxylase (PEPC) in mesophyll cells. Carbon is then shuttled as 4-carbon acids to the bundle sheath cells where CO_2_ is released by a C_4_ acid decarboxylase. Three decarboxylases, NAD-dependent malic enzyme (NAD-ME), NADP-dependent malic enzyme (NADP-ME), and phosphoenolpyruvate carboxykinase (PEPCK), are known to operate in C_4_ plants to release CO_2_ for reassimilation by Rubisco in the Calvin–Benson–Bassham cycle (Hatch 1987; Kagawa and Hatch 1974; Wang, Bräutigam, et al. 2014). The directional transport of organic acids from mesophyll to bundle sheath combined with bundle sheath–preferential accumulation of Rubisco in C_4_ plants ensures that Rubisco operates under high CO_2_ concentrations (Sage et al. 2012).

The recruitment of C_4_ genes from the C_3_ photosynthetic pathway not only required mechanisms that led to patterns of cell-preferential gene expression but also increased transcript levels (Langdale and Nelson 1991; Hibberd and Covshoff 2010). These 2 traits are likely to have evolved independently as they can be controlled by different *cis*-elements in the same gene (Marshall et al. 1997; Akyildiz et al. 2007; Kajala et al. 2012; Wiludda et al. 2012). Moreover, cell-preferential accumulation of C_4_ enzymes can be specified at different levels of regulation (Gowik et al. 2004, 2017; Heimann et al. 2013; Williams et al. 2016). For example, epigenetic regulation has been documented in the C_4_ monocotyledon maize (*Zea mays*) where mesophyll-preferential expression of *CA* and *PEPC* seems to be regulated by trimethylation of histone H3K4 at analogous gene positions (Heimann et al. 2013). Transcriptional control is also important in C_4_ dicotyledons such as *Flaveria bidentis* and *Gynandropsis gynandra*. For example, in *F. bidentis*, mesophyll-preferential expression of *PEPC* is transcriptionally controlled by *cis*-elements known as mesophyll-enhancing module 1 (MEM1) and MEM1-like, respectively (Gowik et al. 2004, 2017), and in *G. gynandra*, bundle sheath–preferential accumulation of NAD-ME1, NAD-ME2, and mitochondrial malate dehydrogenase (MDH) is controlled by a pair of *cis*-elements that, despite being exonic, act transcriptionally (Reyna-Llorens et al. 2018). In *G. gynandra*, posttranscriptional regulation is also important, with for example mesophyll-preferential accumulation of CA and pyruvate,orthophosphate dikinase (PPDK) being determined through the mesophyll expression module 2 (MEM2) found in 5′ and 3′ UTRs (Williams et al. 2016). There is also evidence that translational regulation is important in maintaining cell-specific accumulation of PEPC in maize mesophyll cells and of Rubisco in maize and amaranth (*Amaranthus hypochondriacus*) bundle sheath cells (Berry et al. 1986, 1988; Wostrikoff et al. 2012; Chotewutmontri and Barkan 2021).

Despite progress made in understanding global transcriptomic changes associated with the expression of C_4_ genes between cell types (Chang et al. 2012; John et al. 2014; Ponnala et al. 2014; Aubry et al. 2016), across developmental gradients (Aubry et al. 2014; Külahoglu et al. 2014; Kümpers et al. 2017), and in response to light (Hendron and Kelly 2020), to our knowledge, very little is known about the effect of photoperiod on cell-preferential gene expression in the C_4_ leaf. To address this, we grew maize under controlled conditions, measured photosynthesis, and performed RNA sequencing (RNA-seq) from mesophyll and bundle sheath strands over a 24-h time course. Although growth conditions were constant, rates of photosynthesis and cell-preferential expression of C_4_ genes varied during the photoperiod. In fact, the largest differences in C_4_ cycle transcript abundance between mesophyll and bundle sheath cells were detected between 6 and 10 h after dawn, when rates of C_4_ photosynthesis were highest. By integrating a DNA motif enrichment analysis with a gene coexpression network analysis, we identified transcription factors from DNA binding with one finger (DOF) and MADS (M for MINICHROMOSOME MAINTENANCE FACTOR 1, A for AGAMOUS, D for DEFICIENS and S for Serum Response Factor) families as candidate regulators of bundle sheath–preferential expression. *Trans*-activation assays in planta confirmed the ability of these DOF and MADS transcription factors to activate promoter fragments of the bundle sheath preferential *NADP-ME* and *PEPCK* maize genes.

## Results

### Rates of photosynthesis fluctuate under constant light and temperature

**Figure 1.**
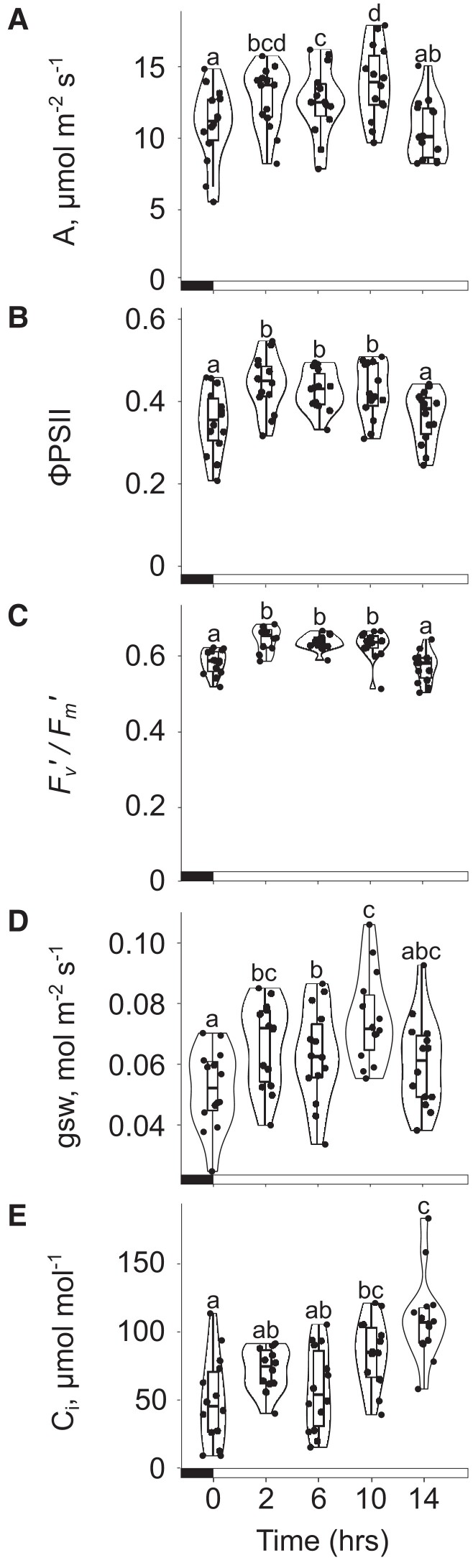
Photosynthetic efficiency in maize fluctuates across the photoperiod. **A to E)** Violin plots and boxplots showing photosynthetic parameters of light-adapted leaves during constant light and temperature. **A)** CO_2_ assimilation (A) rate. **B)** Operating efficiency of PSII (ϕPSII). **C)** Maximum efficiency of PSII photochemistry in the light (*F_v_*′*/F_m_*″). **D)** Stomatal conductance (gsw) to water vapor. **E)** intercellular CO_2_ concentration (C_i_,). Boxplot center line is median, box limits denote the interquartile range, whiskers are minimum and maximum (outliers outside this), and tails indicate 95% confidence intervals and different letters denote statistically significant differences between time points determined by 1-way repeated measures ANOVA, Tukey test (*P* ≤ 0.05, *n* = 14 biological replicates). Each data point represents 1 biological replicate. Black and white bars in the *x* axis denote dark and light periods, respectively.

Photosynthetic parameters of C_4_ maize leaves exposed to constant light and temperature were determined 2, 6, 10, and 14 h after dawn (Fig. 1, A to E). *F_v_/F_m_* values from dark-adapted leaves (Supplemental Table S1) were consistent with those expected from unstressed leaves (Demmig and Björkman 1987). Despite light intensity being constant, statistically significant variations in assimilation rate were detected (Fig. 1A; Supplemental Table S1) with the highest rates occurring 10 h after dawn. The chlorophyll fluorescence parameters ϕPSII and *F_v_*′*/F_m_*″ that report on the operating efficiency of Photosystem II (PSII) and maximum efficiency of PSII without dark adaptation respectively showed slightly different dynamics with values stabilizing from 2 h after dawn (Fig. 1, B and C). Coincident with the variation in carbon fixation, stomatal conductance increased from dawn to 10 h (Fig. 1D). The relative increase in stomatal conductance exceeded that of net CO_2_ fixation, and as a result, the intercellular CO_2_ concentration in the leaf increased consistently over the entire 14 h of light (Fig. 1E). Overall, these data reveal that without alterations in light intensity, photosynthetic parameters in C_4_ maize fluctuate across the day, with higher CO_2_ assimilation at 10 h after dawn (Fig. 1A). The trend of increased CO_2_ assimilation, stomatal conductance, and intercellular concentration of CO_2_ until 10 h after dawn contrasted with ϕPSII and *F_v_*′*/F_m_*″ that peaked after only 2 h of light (Fig. 1, A to E). To initiate a molecular investigation of processes associated with these alterations to C_4_ photosynthesis over the photoperiod, we assessed genome-wide patterns of transcript abundance in mesophyll and bundle sheath cells over a 24-h period.

### Compartmentation of C_4_ cycle gene expression varies during the day

**Figure 2.**
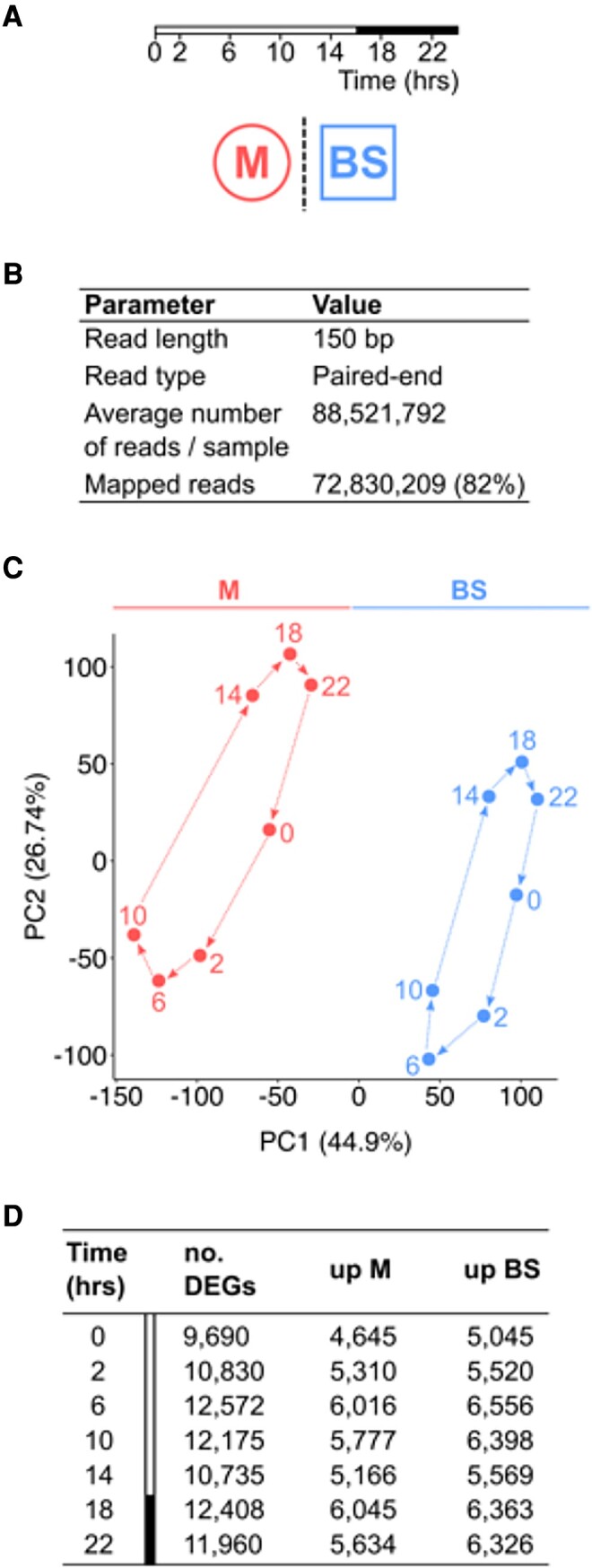
Maize mesophyll and bundle sheath transcriptomes over a diel time course. **A)** Mesophyll and bundle sheath transcriptomes were collected over 24 h. White and black bars denote light and dark periods, respectively. **B)** Transcriptome sequencing parameters. **C)** PCA of mesophyll and bundle sheath transcriptomes. Principal component (PC) 1 and PC2 explain 45% and 27% of data variance, respectively. **D)** Number of differentially expressed genes (DEGs) between mesophyll and bundle sheath cells at each time point: upregulated in mesophyll (log_2_(M/BS) > 0) or bundle sheath (log_2_(M/BS) < 0) (DESeq2 differential expression testing with multiple test-corrected *P*_adj_ < 0.01). M and BS represent mesophyll and bundle sheath cells, respectively.

RNA was isolated from mesophyll and bundle sheath cells over a 24-h period and subjected to deep sequencing. Samples were collected at 0, 2, 6, 10, 14, 18, and 22 h after dawn in a 16-h photoperiod (Fig. 2A). A total of 88,521,792 reads were obtained per sample, of which 82% mapped to the maize reference genome B73 AGPv3 (Fig. 2B; Supplemental Table S2). Quality control for reproducibility showed strong correlation between biological replicates (Pearson's *r* > 0.94; Supplemental Fig. S1). Principal component analysis (PCA) showed that cell type (mesophyll or bundle sheath) accounted for the first principal component and explained 45% of the variance (Fig. 2C). Time of day was associated with the second principal component and accounted for 27% of the variance (Fig. 2C). This implies that transcript abundance in the maize leaf is influenced by both cell type and time of day. To determine whether the spatial patterning of transcripts between mesophyll and bundle sheath cells showed temporal dynamics, differential gene expression analysis was performed at each time point. The maximum number of differentially expressed genes between these cell types (12,572) was detected at 6 h after dawn, while the minimum number (9,690) was observed at dawn (0 h) (Fig. 2D; Supplemental Table S3).

Core components of the maize circadian oscillator changed over the time course as would be expected from analysis of C_3_ species. Maize orthologs for circadian oscillator components were defined using OrthoFinder (Emms and Kelly 2019) using proteomes of *Arabidopsis* (*Arabidopsis thaliana*) (C_3_), *Z. mays* (C_4_), *Oryza sativa* (C_3_), *Triticum aestivum* (C_3_), *Brachypodium distachyon* (C_3_), *Setaria italica* (C_4_), and sorghum (*Sorghum bicolor*) (C_4_) as input (Supplemental Fig. S2A and Table S4). Many circadian oscillator genes in *A. thaliana* had more than 1 ortholog in maize (Supplemental Table S4), consistent with the multiple gene duplications in the maize lineage since it diverged from their last common ancestor (Lee et al. 2013). Specifically, *Arabidopsis* pseudo-response regulator 7 (PRR7, AT5G02810) had 3 orthologs in maize, hereafter referred to as PRR7.1 (GRMZM2G005732), PRR7.2 (GRMZM2G033962), and PRR7.3 (GRMZM2G095727) (Supplemental Fig. S2B). By contrast, *Arabidopsis* PRR3 (AT5G60100), PRR5 (AT5G24470), PRR9 (AT2G46790), and a CCT motif family protein (AT2G46670) were part of the same clade and shared 2 orthologs PRR3/5/9.1 (GRMZM2G179024) and PRR3/5/9.2 (GRMZM2G367834) in maize (Supplemental Fig. S2C). As expected, maize circadian oscillator genes were expressed in temporal waves with *CCA1/LHY.1* and *CCA1/LHY.2* transcripts peaking 6 h after dawn (Supplemental Fig. S2D). The peak in *CCA1/LHY* transcript abundance was followed by sequential accumulation of *PRRs*. For example, transcripts of *PRR7.1* to *PRR7.3* accumulated between 6 and 10 h of light, and *PRR3/5/9.1* and *PRR3/5/9.2* peaked at 10 and 14 h after dawn (Supplemental Fig. S2D). A rise in abundance was then observed for the evening/night transcripts *LUX ARRHYTHMO* (*LUX*) and *TIMING OF CAB EXPRESSION 1* (*TOC1.1* to *TOC1.6*) such that they peaked 2 h after the dark period (Supplemental Fig. S2D). Despite *EARLY FLOWERING 3* (*ELF3*) being an evening component in *A. thaliana* (Nusinow et al. 2011), in maize, *ELF3* transcript abundance was slightly higher during the day (Supplemental Fig. S2D). This observation is consistent with previous observations showing *ELF3* peaking near dawn in sorghum, foxtail millet, rice, and wheat (Zhao et al. 2012; Lai et al. 2020; Wittern et al. 2022). Notably, while most core components of the maize circadian oscillator appeared to be partitioned equally between the 2 cell types, transcripts for *PRR7.1* to *PRR7.3* and *ELF3* were more abundant in bundle sheath cells across the day (Supplemental Fig. S2D).

**Figure 3.**
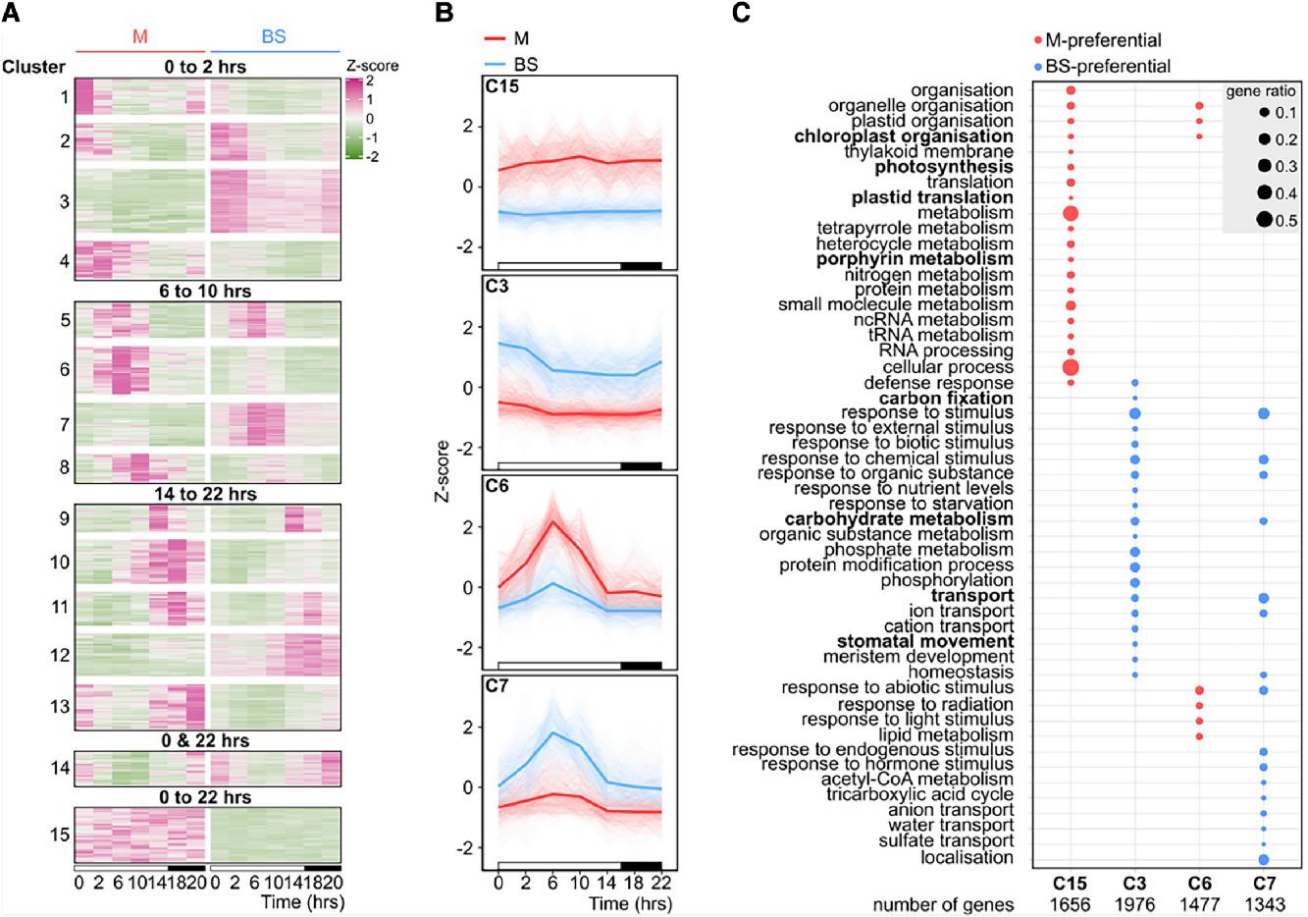
GO terms associated with time of day and cell type in the maize leaf. **A)** Heatmap illustrating profiles of transcript abundance of coexpressed genes in mesophyll and bundle sheath cells across the diel time course. Clusters are grouped based on the time they peak (from dawn to 2 h of light, 6 to 10 h, 14 to 22 h, dawn and 22 h, and dawn to 22 h). *x* axis represents time and *y* axis *Z*-score. High-to-low *Z*-score values are shown as pink to green. **B)** Line plots representing the diel transcript abundance profile of Clusters 15, 3, 6, and 7 in mesophyll and bundle sheath cells across the diel time course. Thick lines denote the mean of *Z*-score values in mesophyll or bundle sheath. The *x* axis represents time points and the *y* axis *Z*-score values. White and black bars in the *x* axis denote light and dark periods, respectively. **C)** Dot plot showing the 20 categories of biological processes with the highest significance for Clusters 15, 3, 6, and 7 (FDR ≤ 0.01). Gene ratio represents the proportion of genes assigned to a functional category in a cluster. M and BS represent mesophyll and bundle sheath cells, respectively.

To investigate whether the circadian clock modulates C_4_ photosynthesis, we measured photosynthetic activity under 1 light–dark cycle followed by 72 h of a light regime that consisted of 40-min light and 20-min darkness (Supplemental Fig. S3). Rhythmic oscillations with near 24-h free-running circadian periods were detected in the chlorophyll fluorescence parameters *F_m_*, *F_v_/F_m_*, ϕPSII, and *F_v_*′*/F_m_*″ that report on the maximum yield of fluorescence, maximum quantum efficiency of PSII photochemistry, operating efficiency of PSII, and maximum efficiency of PSII (empirical *P* < 0.01; Supplemental Fig. S3, A to D). ϕPSII and *F_v_*′*/F_m_*″ (Supplemental Fig. S3, C and D) showed similar dynamics to those observed in the dark–light cycle (Fig. 1, B and C) with higher values occurring between 2 and 10 h after dawn. The 24-h cycle of photosynthetic parameters in these conditions is indicative of circadian regulation. To define groups of genes with maximal transcript abundance at different times of day in each cell type, k-means clustering was performed (Supplemental Table S5). This identified 15 clusters of genes that were divided into 5 groups based on their peak in expression (Fig. 3A; Supplemental Table S5). Of the 15 clusters defined, 3 of them did not show a strong cell-specific profile (Clusters 5, 9, and 11). On the other hand, we observed a clear separation of the clusters defined by the peaks of activity and cell-type–preferential expression for the remaining 12 clusters (Fig. 3A). To better understand these broad alterations in gene expression, gene ontology (GO) enrichment analysis was performed on each cluster (Supplemental Fig. S4 and Table S6). Signaling cascades peaked early in the morning in both cell types. Later on, transcripts associated with chloroplast organization, photosynthesis, and response to light peaked in mesophyll cells, while transport peaked in the bundle sheath. The activation of genes involved in transcription, translation, and protein metabolism was observed during the transition to the dark period (Supplemental Fig. S4 and Table S6).

Clusters 3, 6, 7, and 15 contained transcripts that showed the most distinct differences in expression between mesophyll and bundle sheath cells (Fig. 3B), and so we assessed the nature of genes encoding these transcripts. Cluster 15 contained genes preferentially expressed in the mesophyll throughout the diel time course and was strongly enriched in biological processes such as chloroplast organization, photosynthesis, plastid translation, and porphyrin metabolism (Fig. 3, B and C; Supplemental Table S6). In contrast, Cluster 3 was bundle sheath–preferential and enriched GO terms included carbon fixation, carbohydrate metabolism, transport, and stomatal movement (Fig. 3, B and C; Supplemental Table S6). Interestingly, chloroplast organization was also enriched in Cluster 6 of mesophyll-preferential genes that peaked at 6 h after dawn and Cluster 7 that contained genes involved in carbohydrate metabolism and transport that were bundle sheath preferential (Fig. 3, B and C; Supplemental Table S6).

Consistent with enrichment in the photosynthesis GO term, Cluster 15 contained genes from both the core C_4_ and Calvin–Benson–Bassham cycles (*PEPC*, *ASPARTATE AMINOTRANSFERASE* from mesophyll [*AspAT (M*)] and *PPDK*, and *TRIOSEPHOSPHATE ISOMERASE* [*TPI*]) (Supplemental Table S7). Moreover, Cluster 3 was enriched in C_4_-related genes (*NADP-ME*, *RIBULOSE 1,5-BISPHOSPHATE CARBOXYLASE/OXYGENASE ACTIVASE* [*RCA*], *FRUCTOSE-1,6-BISPHOSPHATASE* [*FBP*], *TRANSKETOLASE* [*TKL*], *RIBULOSE-PHOSPHATE3 EPIMERASE* (*RPE*), *SEDOHEPTULOSE-1,7-BISPHOSPHATASE* [*SBP*], and *PHOSPHORIBULOKINASE* [*PRK*]). This was also the case for Clusters 6 and 7 (with Cluster 6 containing *CA* and *GLYCERALDEHYDE 3-PHOSPHATE DEHYDROGENASE B SUBUNIT* [*GAPDH(B)*] and Cluster 7 containing *PEPCK*; *Rubisco SMALL SUBUNIT-3m* [*RBCS3m*], *GLYCERALDEHYDE 3-PHOSPHATE DEHYDROGENASE A SUBUNIT* [*GAPDH(A)*], and *FRUCTOSE BISPHOSPHATE ALDOLASE* [*FBA*]) (Supplemental Table S7).

**Figure 4.**
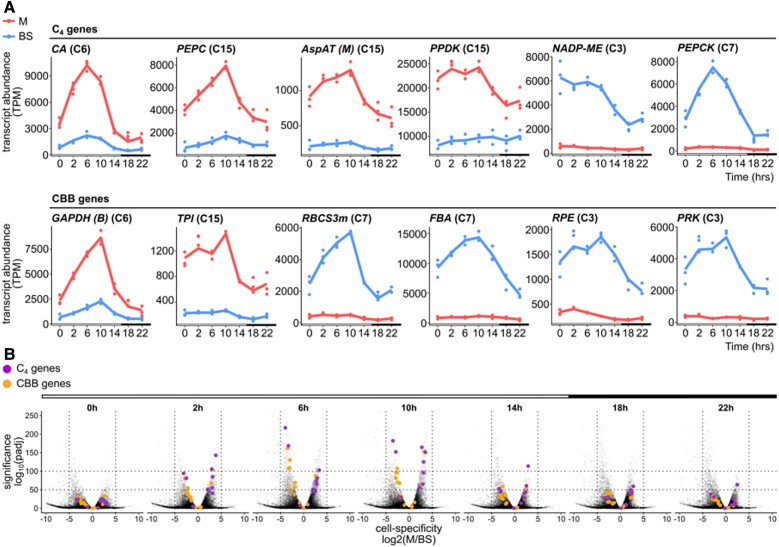
Cell specificity of C_4_ cycle and Calvin–Benson–Bassham (CBB) cycle transcripts oscillates over the time course. **A)** C_4_ genes and CBB cycle genes present in Clusters 15, 3, 6, and 7. *x* axis depicts time and *y* axis shows transcript abundance in TPM. White and black bars denote light and dark periods. Gene names are followed by cluster number in parentheses. **B)** Volcano plots showing the distribution of adjusted *P* values in relation to the fold change between mesophyll and bundle sheath cells. Purple and orange circles denote C_4_ and CBB cycle genes, respectively, and gray data points the remaining transcriptome.

Transcript abundance of C_4_ cycle genes in Clusters 3, 6, 7, and 15 varied over the diel time course and tended to peak during the light period (Fig. 4A). Maximal transcript abundance of most C_4_ cycle and also Calvin–Benson–Bassham cycle genes took place between 6 and 10 h of light (Fig. 4A). Indeed, during the first 10 h of light, there was a gradual increase in the statistical significance associated with the extent to which C_4_ and Calvin–Benson–Bassham cycle transcript abundance was partitioned between mesophyll and bundle sheath cells (Fig. 4B). Taken together, these data reveal a striking variation in the extent to which C_4_ photosynthesis genes are preferentially expressed in mesophyll or bundle sheath cells over the day.

### Members of the DOF and MADS-domain transcription factor families as regulators of bundle sheath–preferential expression of C_4_ and Calvin–Benson–Bassham cycle genes

**Figure 5.**
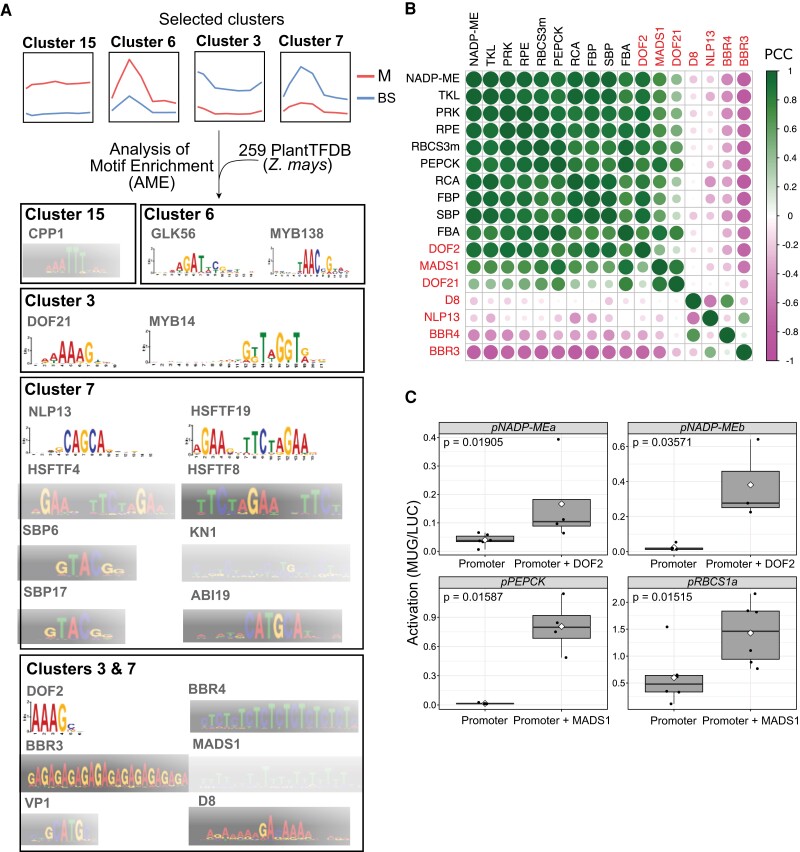
Motifs and transcription factors associated with cell-preferential gene expression. **A)** Four clusters were selected for analysis. DNA-binding motifs enriched in mesophyll Clusters 15 and 6 or bundle sheath Clusters 3 and 7. **B)** Heatmap illustrating Pearson's correlation coefficient (PCC) values for bundle sheath–preferential photosynthesis genes in Clusters 7 and 3 and candidate transcriptional regulators. DOF2, GRMZM2G009406; MADS1, GRMZM2G171365; DOF21, GRMZM2G162749; Dwarf Plant 8 (D8), GRMZM2G144744; NLP13, GRMZM2G053298; BBR4, GRMZM2G118690; BBR3, GRMZM2G164735. **C)** Line plots of diel transcript abundance for candidate regulators of bundle sheath–preferential photosynthesis genes. *x* axis shows time and *y* axis *Z*-score. White and black bars in the *x* axis denote light and dark periods, respectively. M and BS represent mesophyll and bundle sheath cells. MADS1, GRMZM2G171365; DOF21, GRMZM2G162749; DOF2, GRMZM2G009406; BBR3, GRMZM2G164735; BBR4, GRMZM2G118690. **D)** Box plots showing promoter activation of bundle sheath–preferential genes *NADP-ME* (Cluster 3), *PEPCK* (Cluster 7), and *RBCS* (Cluster 7) by transcription factors DOF2 and MADS1. Black dots denote the individual data points and white diamonds represent the mean. The middle line in each boxplot denotes the median, with the box showing the interquartile range and the whiskers the maximum and minimum. *P* values were obtained from 2-sided, pairwise *t*-tests. *n* = 6 for *pNADMEa*, *pRBCS*, and *pRBCS* + MADS1, *n* = 5 for *pNADPMEb* and *pPEPCK*, *n* = 4 for *pNADMEa* + DOF2 and *pPEPCK* + MADS1, and *n* = 3 for *pNADMEb* + DOF1.

We next sought to use the RNA-seq time course to identify *cis*-elements and *trans*-factors linked to the control of C_4_ gene expression. Thus, to identify potential regulators in *cis* and *trans* of genes in clusters associated with the C_4_ and Calvin–Benson–Bassham cycles (Clusters 15, 6, 3, and 7), we performed a motif enrichment analysis using a set of 259 DNA-binding motifs for *Z. mays* from the PlantTFDB (Jin et al. 2017; Fig. 5A; Supplemental Fig. S5 and Table S8). Of the motifs tested, less than 10% were enriched in at least 1 of the 4 clusters (Fisher's exact test, *P* < 0.01; Supplemental Fig. S5 and Table S8). Mesophyll-preferential clusters were enriched in only 3 motifs. While Cluster 15 was enriched in the CPP-transcription factor 1 (CPP1) motif, Cluster 6 was enriched in G2-like-transcription factor 56 (GLK56) and MYB-transcription factor 138 (MYB138) motifs (Fig. 5A; Supplemental Fig. S5). The GLK56 transcription factor is a known regulator of the circadian clock (Zhao et al. 2023), activating *CCA1* and being coregulated with TOC1. *GLK56* expression peaked at 18 h similar to *TOC1* orthologs (Supplemental Figs. S2D and S5). However, bundle sheath–preferential clusters showed a higher number of enriched motifs. Cluster 3 was enriched in DNA-binding one zinc finger 21 (DOF21) and MYB-transcription factor 14 (MYB14) motifs, while Cluster 7 showed enrichment in NLP-transcription factor 13 (NLP13), KNOTTED 1 (KN1), ABI3-VP1-transcription factor 19 (ABI19), and several members of the HSF and SBP transcription factor families (Fig. 5A). Moreover, both clusters shared an enrichment for a pair of BBR/BCP-transcription factor (BBR) motifs (BBR3 and BBR4) and motifs recognized by DNA-binding one zinc finger 2 (DOF2) and MADS-domain protein 1 (MADS1) (Fig. 5A; Supplemental Fig. S5). This finding suggests that these transcription factors might contribute to bundle sheath–preferential gene expression across the day.

To further investigate links between enriched motifs and photosynthesis genes present in Clusters 15, 6, 3, and 7, a gene coexpression network was built between the corresponding transcription factors and photosynthesis genes containing motif hits (Supplemental Fig. S6). Although we started with the 4 clusters associated with either mesophyll or bundle sheath strands, for 2 reasons, we focused on those defined by bundle sheath–preferential expression (Clusters 3 and 7). First, we did not detect any motif hits for photosynthesis genes present in mesophyll Cluster 6. Second, poorly expressed transcription factors (transcripts per million [TPM] reads < 5) were removed, and this meant that photosynthesis genes from Cluster 15 were also no longer present in the network (Supplemental Fig. S6). Pearson's correlation coefficient was used to define negative or positive coexpression between bundle sheath–preferential photosynthesis genes and candidate transcriptional regulators (Fig. 5B). DOF2, MADS1, and DOF21 were positively coexpressed with bundle sheath–preferential photosynthesis genes in Cluster 3 (*NADP-ME*, *TKL*, *PRK*, *RPE*, *RCA*, *FBP*, *SBP*, and *FBA*) and Cluster 7 (*RBCS3m* and *PEPCK*), while BBR4 and BBR3 showed negative coexpression correlation with these photosynthesis genes (Fig. 5B; Supplemental Table S8). These relationships are underpinned by *MADS1* and *DOF21* being preferentially expressed in bundle sheath cells and peaking 6 h after dawn, while *BBR3* and *BBR4* peaked toward the end of the light period and were preferentially expressed in mesophyll cells (Fig. 5C). We therefore hypothesized that MADS1 and DOF21 act as positive transcriptional regulators of bundle sheath expressed genes while BBR3 and BBR4 act to repress these genes in mesophyll cells. To initiate testing, a *trans*-activation assay in *Nicotiana benthamiana* was performed. Promoter fragments from the *NADP-ME*, *RBCS*, and *PEPCK* genes containing the relevant motifs generated low levels of autoactivation (Fig. 5D), and so we were not able to test for negative regulation by BBR3 and BBR4. However, the DOF2 and MADS1 transcription factors activated short promoter fragments of the *NADP-ME*, *PEPCK*, and *RBCS* genes containing their cognate motifs (Fig. 5D). The combined findings that DOF2 and MADS1 are coexpressed with C_4_ genes, that their DNA binding sites are found in C_4_ promoters, and that they *trans*-activate expression in planta indicate that these transcription factor families likely play a role in enhancing C_4_ gene expression in the bundle sheath during the day.

## Discussion

### Variation in the rate of C_4_ photosynthesis over the day is influenced by circadian oscillations

Our analysis shows that under moderate illumination and a constant light regime similar to those used in *A. thaliana*, barley, and wheat to study circadian oscillations (Dakhiya et al. 2017; Litthauer et al. 2015, 2016; Wittern et al. 2022), photosynthetic rates vary in maize. These findings are therefore consistent with the fact that photosynthesis in C_3_ species is modulated by the circadian oscillator (Dodd et al. 2005) and our analysis of chlorophyll fluorescence quenching in maize supports this notion. The circadian oscillator also regulates stomatal conductance in C_3_ and C_4_ leaves (Resco de Dios and Gessler 2018). Consistent with the circadian regulation of stomatal conductance and photosynthetic efficiency as has been reported in C_3_ species (Harmer et al. 2000; Dodd et al. 2005) 14 h after dawn, all photosynthetic parameters except intercellular concentration of CO_2_ appeared to decline. In this study, CO_2_ assimilation and stomatal conductance followed a different trajectory compared with ϕPSII and *F_v_*′*/F_m_*″, with the former reaching maximum values at 10 h and the latter at 2 h after dawn. This apparent increase in rates of CO_2_ assimilation during the day compared with activity of the photosystems could be because the carbon concentrating mechanism operating in maize is not completely CO_2_ saturated before 10 h. If this is the case, stomatal opening over the day would allow increased intercellular concentration of CO_2_ and thus higher CO_2_ assimilation. It is also possible that the efficiency of carbon assimilation rises during the day and stomata respond to this to maintain CO_2_ supply. A third possibility is that at dawn, C_4_ photosynthesis operates exclusively with NADP-ME for decarboxylation. As the day progresses, the sustained activity of PSII provides sufficient NADPH in the bundle sheath for PEPCK to act as a second decarboxylase. These hypotheses could be mediated by modifications to the transcriptional activity of genes involved in the C_4_ pathway.

### Compartmentation of C_4_ gene expression between mesophyll and bundle sheath varies during the day

Over the light and dark period, we detected statistically significant variance in transcript abundance in mesophyll and bundle sheath strands. Although the main factor explaining this was associated with preferential accumulation of transcripts to either the mesophyll or bundle sheath, time of day also had a significant effect. Thus, although C_4_ cycle transcripts are differentially expressed between the 2 cell types (Chang et al. 2012; Li et al. 2010; Tausta et al. 2014), this compartmentation is more dramatic at midday prior to the highest rates of photosynthesis.

Differences in transcript abundance between the 2 cell types were associated with the mesophyll being biased toward strong expression of components of the photosynthetic electron transport chain as well as responses to far red, red, and blue light. In contrast, GO terms overrepresented in the bundle sheath were involved in carbon fixation and transport. These findings are consistent with previous knowledge relating to the different roles of mesophyll and bundle sheath cells in C_4_ plants. For example, it is known that maize mesophyll cells contain both Photosystems I and II while bundle sheath strands contain Rubisco and fail to accumulate substantial amounts of PSII (Meierhoff and Westhoff 1993). Not only did transcripts encoding components of the core photosynthetic apparatus vary in the extent to which they were compartmented between mesophyll and bundle sheath cells, but this was also the case for transcripts associated with signal transduction pathways and stomatal movement. In both cases, their transcripts tended to peak prior to those associated with carbon fixation. By studying the dynamics of C_4_ photosynthesis throughout the diel cycle, we provide insight into the dynamics of transcript abundance in mesophyll and bundle sheath strands of a C_4_ species. In the C_3_ crop rice, transcriptomics indicated that the bundle sheath is conditioned for roles in water transport, sulfur assimilation, and jasmonic acid biosynthesis (Hua et al. 2021). Employing a similar approach to the 1 reported here would test whether the compartmentation of gene expression underpinning these processes is dynamic across the diel cycle in the rice bundle sheath.

### The role of MADS-domain and DOF transcription factors in activation of C_4_ genes in the bundle sheath

In addition to biological processes being enriched in either mesophyll or bundle sheath strands and the extent of this being time of day dependent, we observed spatiotemporal changes to transcripts encoding multiple transcription factor families. To better understand how transcriptional regulators control the expression of C_4_ and Calvin–Benson–Bassham cycle genes, we performed a motif enrichment analysis on photosynthesis genes followed by a gene coexpression analysis between photosynthesis genes that showed enrichment in DNA-binding motifs and their target transcription factors. This predicted that shared *cis*-elements and *trans*-factors control bundle sheath specificity of genes from both the C_4_ and Calvin–Benson–Bassham cycles, which might ensure spatial and temporal coordination between these 2 photosynthetic cycles. We did not find motifs that could explain the expression patterns of genes encoding components of the light-dependent reactions of photosynthesis as well as the C_4_ and Calvin–Benson–Bassham cycles. This suggests that multiple regulators control different subsets of the photosynthetic apparatus.

Although, to our knowledge, the specific *cis*-elements and transcription factors identified here have not previously been implicated in controlling C_4_ photosynthesis, there are several reports showing that multiple C_4_ genes can be regulated by the same process. For example, mesophyll-specific expression of *PEPC* and *CA* in *F. bidentis* and bundle sheath–specific expression of *NAD-ME1*, *NAD-ME2*, and mitochondrial *MDH* in *G. gynandra* are regulated by pairs of *cis*-elements with high sequence similarity (Gowik et al. 2004, 2017; Reyna-Llorens et al. 2018). Moreover, *PEPC* and *CA* are coordinately regulated by trimethylation of histone H3K4 (Heimann et al. 2013). A comparative analysis of transcriptomes from rice and maize leaf developmental gradients predicted 118 transcription factors as candidate regulators of C_4_ gene expression (Wang, Czedik-Eysenberg, et al. 2014). Among these, ZmMYB138 and ZmSBP6 were also predicted by our pipeline to regulate mesophyll-preferential and bundle sheath–preferential clusters of genes respectively. Our analysis also identified 3 positive (ZmDOF2, ZmMADS1, and ZmDOF21) and 2 negative regulators (ZmBBR3 and ZmBBR4) as strong candidates for determining preferential expression of photosynthesis genes in the bundle sheath. In the analysis of rice and maize transcriptomes (Wang, Czedik-Eysenberg, et al. 2014), DOF-binding *cis*-elements (WAAAG; W = T/A) were also enriched in bundle sheath–specific genes, and it was proposed that they have been recruited from the ancestral C_3_ state to drive bundle sheath–specific expression. Different predictions from the 2 studies are likely explained by the nature of the transcriptomic data sets used. For example, it is possible that analysis of transcriptomes from rice and maize (Wang, Czedik-Eysenberg, et al. 2014) identified regulators that establish differences between the C_3_ and C_4_ systems, whereas the sampling strategy in our case was able to predict genes that maintain and fine-tune cell-preferential gene expression over the photoperiod. In maize, the C_4_ acid decarboxylases NADP-ME and PEPCK drive malate and aspartate metabolism in bundle sheath cells as sources of CO_2_ for Rubisco in the Calvin–Benson–Bassham cycle (Chang et al. 2012; Li et al. 2010; Tausta et al. 2014). Our understanding of how *NADP-ME* and *PEPCK* genes are transcriptionally regulated in C_4_ plants is limited. To date, only ZmbHLH128 and ZmbHLH129 were shown to bind the maize *NADP-ME* promoter in vivo (Borba et al. 2018; Schlüter and Weber 2020). Our pipeline identified DOF2 as a candidate activator of diel and bundle sheath–preferential expression of *NADP-ME* and MADS1 as an activator of *PEPCK* and *RBCS*. Transactivation assays confirmed interaction between these transcription factors and promoters of the C_4_ genes in planta. Notably, DOF2 in maize has previously been shown to repress transcription of the C_4_*PEPC* gene (Yanagisawa 2000; Yanagisawa and Sheen 1998). Our findings therefore suggest that maize DOF2 plays a dual function in the regulation of C_4_ genes in bundle sheath cells through repression of *PEPC* and activation of *NADP-ME.* A more thorough characterization of both DOF2 and MADS1 transcription factors including the generation of mutant alleles is required to confirm their involvement in modulating C_4_ photosynthesis in vivo. Despite transcription factors often being classified as “activators” or “repressors,” some can have both roles depending on the *cis*-regulatory element to which they bind the structure of the surrounding chromatin, protein posttranslational modifications, and interaction with other proteins (Boyle and Després 2010).

The work reported here extends our understanding of C_4_ regulation. For example, the diel and spatial patterning of *RBCS* in C_4_ is well characterized and known to be controlled by multiple levels of gene regulation, including transcriptional and posttranscriptional (Berry et al. 1986; Giuliano et al. 1988; Borello et al. 1993; Xu et al. 2001; Patel et al. 2004, 2006). In maize, the *RBCS* gene is transcriptionally regulated by 2 independent *cis*-elements present in UTRs. In the 5′ UTR, an I-box is essential for light-mediated activation (Giuliano et al. 1988), while in the 3′ UTR, a HOMO motif, which binds the transcription repressor-maize 1 protein, drives mesophyll repression (Xu et al. 2001). The data presented here identify MADS1 as an additional regulatory element associated with the diel expression of *RBCS*. It seems likely that MADS1 activates *RBCS* gene expression in bundle sheath cells as both are positively coexpressed with *MADS1* and RBCS peaking at 6 and 10 h after dawn, respectively. Combined with previous findings, our data therefore suggest that bundle sheath–preferential expression of *RBCS* is achieved through HOMO-mediated repression of *RBCS* transcription in mesophyll (Xu et al. 2001) combined with MADS1-mediated activation of *RBCS* in bundle sheath.

More broadly, our findings are consistent with previous knowledge that MADS-domain transcription factors are key components of genetic regulatory networks involved in plastic developmental responses in plants (Castelán-Muñoz et al. 2019). MADS1 also enhanced expression of *PEPCK*, and so it seems likely that, as with *RBCS*, *PEPCK* requires additional regulatory elements to allow modulation of cell-preferential gene expression and induction by light. In summary, we report that, in maize, the extent to which C_4_ genes are expressed in either mesophyll cells or bundle sheath strands varies during the day. The distinct dynamics of transcript abundance between the 2 cell types allowed us to undertake a gene coexpression analysis that together with *trans*-activation assays in planta showed that DOF2 and MADS1 act as transcriptional activators of diel and bundle sheath–preferential expression of C_4_ genes. It was also noticeable that cell-preferential expression of C_4_ genes either preceded or was coincident with maximum rates of photosynthesis. Our results demonstrate a clear correlation between transcript abundance and photosynthetic activity throughout the diel time course. However, we cannot dismiss the possibility that additional layers of regulation, for example at the ribosome or posttranslation, contribute to the optimization of C_4_ photosynthesis over the day.

## Materials and methods

### Growth conditions and photosynthetic measurements

Maize (*Z. mays* L.) var. B73 plants were grown in M3 High Nutrient soil (Levington Advance) fertilized with 1 g L^−1^ Osmocote, under 16-h light photoperiod, constant temperature of 26 °C during the day and night, 55% relative humidity and ambient CO_2_ concentration. Plants were grown in controlled environment chambers with a ∼500 *µ*mol m^−2^ s^−1^ photosynthetic photon flux density. A combination of fluorescent and halogen lamps was used to provide light for the transcriptomic experiment (spectrum; λp = 544 nm, λpV = 848.1 mW m^−2^) and light-emitting diodes used for other experiments (spectrum; λp = 591 nm, λpV = 726.3 mW m^−2^). Fully expanded third leaves of 10-d-old maize plants were used for all analyses.

CO_2_ assimilation and chlorophyll fluorescence of 14 10-d-old maize third leaves were measured simultaneously with a portable gas exchange system LI-6800 (LI-COR Biosciences) equipped with a Fluorometer head 6800-01 A (LI-COR Biosciences). Leaves were first equilibrated at 400-ppm CO_2_, an irradiance of 500 *µ*mol m^−2^ s^−1^, red–blue actinic light (90%/10%), leaf temperature 25 °C, 15 mmol mol^−1^ H_2_O, and a flow rate 500 *µ*mol s^−1^. Effective quantum yield of PSII (ϕPSII) was probed simultaneously with the gas exchange measurements under red–blue actinic light (90%/10%) using a multiphase saturating flash routine (Loriaux et al. 2013) with Phases 1 and 3 at 8,000 *µ*mol m^−2^ s^−1^. Maize leaves were dark adapted for 4 h prior to obtaining *F_o_* and *F_m_*, the minimal and maximal levels of fluorescence, respectively.

For measurements of chlorophyll fluorescence in diel and constant light (temperature was set to be at 26 °C in both day and night/subjective night), fragments of 6 10-d-old maize third leaves were excised and placed into individual wells of a black 96-well imaging plate (Greiner) filled with of 0.8% (w/v) bactoagar, ½ MS, and 0.5 *µ*m 6-benzyl-aminopurine adjusted to pH5.7 with 0.5 m KOH and 0.5 m HCl. The plate of leaf fragments was then moved to a CFimager (Technologica Ltd) and allowed to acclimate under 100 *µ*mol m^−2^ s^−1^ blue light until dusk when lights were switched off. At dawn of the following day, a light regime was used to capture “day” images, which consisted of 20-min darkness and 800-ms saturating pulse of 6,172 *µ*mol m^−2^ s^−1^ blue light and 40-min blue light at irradiance 100 *µ*mol m^−2^ s^−1^ and 800-ms saturating pulse of 6,172 *µ*mol m^−2^ s^−1^ blue light, which was repeated every hour. After 16 h, the blue light source was switched off and a single 800-ms saturating pulse of 6,172 *µ*mol m^−2^ s^−1^ blue light was applied once per hour to capture “night” images. At dawn of the next day, this repeating light regime was run continuously for a further 72 h to simulate constant light but with dark breaks to allow imaging as has been used previously (Wittern et al. 2023). Chlorophyll fluorescence parameters were calculated using the image scripts provided by the manufacturer. The empirical *P* values and free-running period estimates associated with each parameter were calculated from linear detrended data collected between time points 48 to 96 h in repeating light using the meta.meta function in the MetaCycle R-package (Wu et al. 2016).

### Mesophyll and bundle sheath strand isolation, RNA extraction, and sequencing

Fully expanded segments of 10-d-old maize third leaves were harvested at 0, 2, 6, 10, 14, 18, and 22 h across the photoperiod. The top 0.5 cm of each leaf was discarded, and the midrib was removed. Mesophyll extracts were isolated as described previously by Covshoff et al. (2013) and bundle sheath strands according to Markelz et al. (2003) and John et al. (2014). Three replicates of 6 leaves each were initially rolled to extract mesophyll sap and then blended to isolate bundle sheath strands. Mesophyll sap was rapidly collected and deposited into RLT lysis buffer for RNA extraction (RNeasy Plant Mini Kit, Qiagen). Excess moisture was removed from the purified bundle sheath strands on a bed of paper towels. Bundle sheath strands were flash frozen in liquid nitrogen and stored at −80 °C prior to RNA extraction.

Total RNA was extracted from 3 independent samples of mesophyll-enriched and bundle sheath–enriched tissues collected at 7 time points (42 samples) using RNeasy Plant Mini Kit (Qiagen). To eliminate residual genomic DNA, the RNA was treated with TURBO DNA-free Kit (Ambion) following the manufacturer's instructions. Initial quality control of total RNA was performed by a photometric measurement on a NanoDrop 1000 device. This was followed by RQN determination via a Fragment Analyzer System (AATI) using the DNF-471 standard sensitivity RNA Assay. Final RNA quantification was performed by a fluorometric Qubit assay (RNA HS, Thermo Fisher Scientific). Library preparation was carried out on a PerkinElmer Sciclone NGS robotics unit using the Illumina TruSeq stranded mRNA sample Preparation Kit (#15031047 Rev.E) following the manufacturer's instructions. Input amount of total RNA was 200 ng. Final libraries were passed through an additional bead clean-up step in a 1:1 ratio (sample/beads) to remove primer dimers. Quality control on a Fragment Analyzer System (AATI) was used to determine fragment length distribution using the DNF-474 Assay. For quantification purposes, a fluorometric Qubit dsDNA HS Assay Kit was used. Libraries were diluted to 2 nm prior to equimolar pooling into 6 separate pools, which were then each sequenced on individual flow cell lanes. Paired-end sequencing with a 2 × 150-bp read length was performed on an Illumina HiSeq3000 system using the HiSeq 3000/4000 PE Cluster Kit (PE-410-1001) and the HiSeq 3000/4000 SBS Kit 300 cycles (FC-410-1003). Clustering and sequencing were carried out following the manufacturer's instructions. Library preparation and sequencing were done at the Genomics and Transcriptomics Laboratory of the University of Düsseldorf.

### Read assembly, annotation, and quantification of transcript abundance

Reads were mapped to the *Z. mays* B73 genome AGPv3 (from Ensembl Plants, http://plants.ensembl.org) and quantified as TPM (Supplemental Table S2) (Wagner et al. 2012) using RSEM version 1.2.23 with default settings (Li and Dewey 2011) in conjunction with Bowtie 1 (Langmead et al. 2009). Differential expression analysis was performed using the DESeq2 R package (Love et al. 2014) with read counts used as input. Cell type was treated as condition (mesophyll vs. bundle sheath). Benjamini–Hochberg corrected *P* value was set to <0.01 to identify differentially expressed genes (Supplemental Table S3) (Benjamini and Hochberg 1995).

### Data analysis and visualization

Data analysis was performed using R (R Development Core Team 2009) unless stated otherwise. The R package ggplot2 (Wickham 2009) was used to generate all graphs. PCA was performed on the mean of transcriptome triplicates of mesophyll and bundle sheath samples collected at 0, 2, 6, 10, 14, 18, and 22 h. Pearson's correlation coefficient was calculated between transcriptomes of 3 biological replicates from mesophyll and bundle sheath samples. K-means clustering was performed on expressed genes (TPM > 5). Genes were quantile normalized and transformed to *Z*-score values. A total of 15 centers were selected based on the total within the sum of squares. GO term enrichment analysis was performed using AgriGO v2 (GO analysis toolkit and database for agricultural community; Tian et al. 2017) with the following settings: statistical test method, Fisher; multitest adjustment method, Hochberg (false discovery rate [FDR]); and GO type, complete GO. A FDR cutoff of ≤0.01 was set to identify significantly enriched GO terms in clusters of coexpressed genes (detailed in Supplemental Table S5).

Genes encoding maize transcription factors were downloaded from PlantTFDB v4.0 (2,331 genes, http://planttfdb.cbi.pku.edu.cn; Jin et al. 2017) and Grassius (2,605 genes, http:://www.grassius.org; Yilmaz et al. 2009). Only the 2,110 genes present in both databases were considered in further analyses. Maize genes encoding transcription factors were assigned into families according to PlantTFDB v4.0. Motif enrichment analysis across genes was performed for each cluster using the “Analysis of Motif Enrichment” tool from the MEME suite (Bailey et al. 2009; McLeay and Bailey 2010) using default parameters. For each transcript present in a particular cluster, promoter sequences (−2 to +0.5 kb from the transcription start site) were retrieved and used as input. Control sequences were defined as the entire set of sequences (all clusters) minus those sequences present in the cluster of interest. A gene coexpression network was built from normalized RNA-seq data and DNA motif enrichment analysis. Transcripts encoding transcription factors with DNA-binding motif hits in photosynthesis genes (both C_4_ and Calvin–Benson–Bassham cycle genes) were filtered by expression levels such that TPM reads were greater than 5. Pearson’s correlation coefficients were then calculated between transcription factors and their potential target genes using a cutoff to define putative interactions of coefficients between <0.3 and >−0.3. Cytoscape (Shannon et al. 2003) was then used to visualize the resulting network (Supplemental Fig. S6).

Maize orthologs were identified for circadian clock genes from *Arabidopsis* (*A. thaliana*) using OrthoFinder (Emms and Kelly 2019) including the proteomes of 7 representative plant species (*A. thaliana*, rice [*O. sativa*], wheat [*T. aestivum*], *B. distachyon*, *S*. *italica*, *S. bicolor*, and maize [*Z. mays*]). Proteomes were downloaded from the ENSEMBL website (www.ensembl.com). Phylogenetic trees were generated using Dendroscope (www.dendroscope.org; Huson et al. 2009).

### *Trans*-activation assays in planta

Constructs were generated using Golden Gate cloning as described in Supplemental Table S9. Coordinates of the *NADP-ME* (GRMZM2G085019) and *PEPCK* (GRMZM2G001696) promoters (1.5-kb upstream of the translation start site) enriched in DOF2 and MADS1 motifs, respectively, were retrieved from the motif enrichment analysis. For the *trans*-activation assays with the *NADP-ME* promoter, 2 fragments of 106 bp that contain a 6-bp DOF2 motif (“aaagcc” in NADP-MEa and “ggcttt” in NADP-MEb) flanked by 50-bp endogenous promoter sequence either side of the motif were cloned upstream of a minimal 35S promoter (Supplemental Table S9). For the *PEPCK* promoter, 1 fragment of 121 bp that contain a 21-bp MADS1 motif (“tttctttcttttgttctccgc”) flanked by 50-bp endogenous promoter sequence either side of the motif was cloned upstream of a minimal 35S promoter (Supplemental Table S9). For the *RBCS* promoter, 1 fragment of 121 bp that contained a 21-bp MADS1 motif (“aaacgaaaaaaataacaaaca”) flanked by 50-bp endogenous promoter sequence either side of the motif was cloned upstream of a minimal 35S promoter (Supplemental Table S9): 106-bp *pNADP-MEa*: −586- to −692-bp upstream of the translation start site with the 6-bp DOF2 motif (“aaagcc”) at −636- to −642-bp upstream of the translation start site; 106-bp *pNADP-MEb*: −112- to −218-bp upstream of the translation start site with the 6-bp DOF2 motif (“ggcttt”) at −162- to −168-bp upstream of the translation start site; 121-bp *pPEPCK*: −815- to −936-bp upstream of the translation start site with the 21-bp MADS1 motif (“tttctttcttttgttctccgc”) at −865- to −886-bp upstream of the translation start site; 121-bp *pRBCS*: −1,089- to −968-bp upstream of the translation start site with the 21-bp MADS1 motif (“aaacgaaaaaaataacaaaca”) at −1,039- to −1,018-bp upstream of the translation start site. Level 1 constructs were made such that these promoter fragments were placed upstream of the GUS reporter gene. To produce Level 2 constructs, these were combined with a transformation control containing the LUCIFERASE (LUC) reporter driven by the constitutive *NOS* promoter, the transcription factor of interest driven by the constitutive *LjUBI* promoter and the P19 silencing suppressor under control of the *CaMV35S* promoter. Constructs were transformed into the *Agrobacterium tumefaciens* strain GV3101. Overnight cultures of *A. tumefaciens* were pelleted and resuspended in infiltration buffer (10 mm MES [pH 5.6], 10 mm MgCl_2_, and 150 *µ*m acetosyringone) to an optical density of 0.3. Cultures were then incubated for 2 h at room temperature and infiltrated into the abaxial side of leaves of 4-wk-old *N. benthamiana* plants with 1-mL syringe. Leaf discs from the infiltrated regions were sampled 48 h after infiltration and flash frozen in liquid nitrogen. Protein for the 4-methylumbelliferyl-b-d-glucuronide (MUG) and LUC assays was extracted in 1× passive lysis buffer (PLB: Promega). MUG assays were performed by adding 40 mL of protein extract to 100 mL of MUG assay buffer (2 mm MUG, 50 mm NaH_2_PO_4_/Na_2_ HPO_4_ buffer [pH 7.0], 10 mm EDTA, 0.1% [v/v] Triton X-1000, 0.1% [w/v] sodium lauroyl sarcosinate, and 10 mm DTT). Stop buffer (200 mm Na_2_CO_3_) was added at 0 and 120 min, and the rate of MUG accumulation was measured in triplicate on a plate reader (CLARIOstar, BMG lab tech) with excitation at 360 nm and emission at 465 nm. LUC activity was measured with 20 mL of protein sample and 100 mL of LUC assay reagent (Promega). Promoter activation was calculated as (rate of MUG accumulation/LUC luminescence) × 100.

### Accession numbers

All referenced gene names and accessions are detailed in Supplemental Tables S2 to S5 and S7. RNA-seq data generated in this study have been deposited to the National Center for Biotechnology Information Sequence Read Archive with accession number PRJNA635519.

## Supplementary Material

kiad447_Supplementary_Data

## Supplemental data

The following materials are available in the online version of this article.

**Supplemental Figure S1.** Heatmap of Pearson's correlation coefficient (PCC) calculated between transcriptomes of biological replicates 1, 2, and 3 of mesophyll (M) and bundle sheath (BS) samples collected at time points 0, 6, 10, 14, 18, and 22 hr.

**Supplemental Figure S2.** Components of the maize circadian oscillator.

**Supplemental Figure S3.** Photosynthetic parameters measured for maize leaf fragments under 1 light–dark cycle (16-h light, 8-h dark) followed by 72 h of a light regime that consisted of cycles of 40-min light and 20-min darkness.

**Supplemental Figure S4.** Distribution of biological processes across the diel time course and between mesophyll and bundle sheath cells.

**Supplemental Figure S5.** Line plots representing the diel transcript abundance profile of genes encoding the cognate transcription factors for DNA-binding motifs enriched in mesophyll-preferential and bundle sheath–preferential clusters of coexpressed genes.

**Supplemental Figure S6.** Gene coexpression network built from RNA-seq data and DNA motif enrichment analysis.

**Supplemental Table S1.** Photosynthetic parameters of dark-adapted maize leaves. *A*, CO_2_ assimilation rate. *F_v_*/*F_m_*, maximum quantum efficiency of PSII photochemistry. gsw, stomatal conductance.

**Supplemental Table S2.** TPM reads obtained from M (mesophyll) and BS (bundle sheath) samples at 0, 2, 6, 10, 14, 18, and 22 h.

**Supplemental Table S3.** Differentially expressed genes between mesophyll and bundle sheath cells at each time point (DESeq2 differential expression testing with multiple test-corrected *P*_adj_ < 0.01).

**Supplemental Table S4.** Orthogroups.

**Supplemental Table S5**. Clusters of coexpressed genes.

**Supplemental Table S6.** GO term enrichment analysis on clusters of coexpressed genes (FDR ≤ 0.01).

**Supplemental Table S7.** Photosynthesis genes in maize.

**Supplemental Table S8.** Maize DNA-binding motifs enriched in Clusters 15, 6, 3, and 7.

**Supplemental Table S9.** *Trans*-activation constructs.

## Dive Curated Terms

The following phenotypic, genotypic, and functional terms are of significance to the work described in this paper:

PPDK Gramene: AT4G15530
PPDK Araport: AT4G15530

TOC1 Gramene: AT5G61380
TOC1 Araport: AT5G61380

MYB14 Gramene: AT2G31180
MYB14 Araport: AT2G31180

PRK Gramene: AT1G32060
PRK Araport: AT1G32060

RCA Gramene: AT2G39730
RCA Araport: AT2G39730

TKL Gramene: AT3G63180
TKL Araport: AT3G63180

CCA1 Gramene: AT2G46830
CCA1 Araport: AT2G46830

MADS1 Gramene: GRMZM2G171365
MADS1 Araport: GRMZM2G171365

MDH Gramene: Q08062
MDH Araport: Q08062

PRR7 Gramene: AT5G02810
PRR7 Araport: AT5G02810

ELF3 Gramene: AT2G25930
ELF3 Araport: AT2G25930

MES CHEBI: CHEBI:39010

NADP-ME Gramene: E9NQE2
NADP-ME Araport: E9NQE2
