# Supplementary material for: Compartmentation of photosynthesis gene expression in C4 maize depends on time of day
Source: Plant Physiol. 2023 Aug 9;193(4):2306–20. doi: 10.1093/plphys/kiad447 (PMC10663113; doi:10.1093/plphys/kiad447)
Supplement: kiad447_Supplementary_Data [file kiad447_supplementary_data.zip › S Figures.v3.pdf]

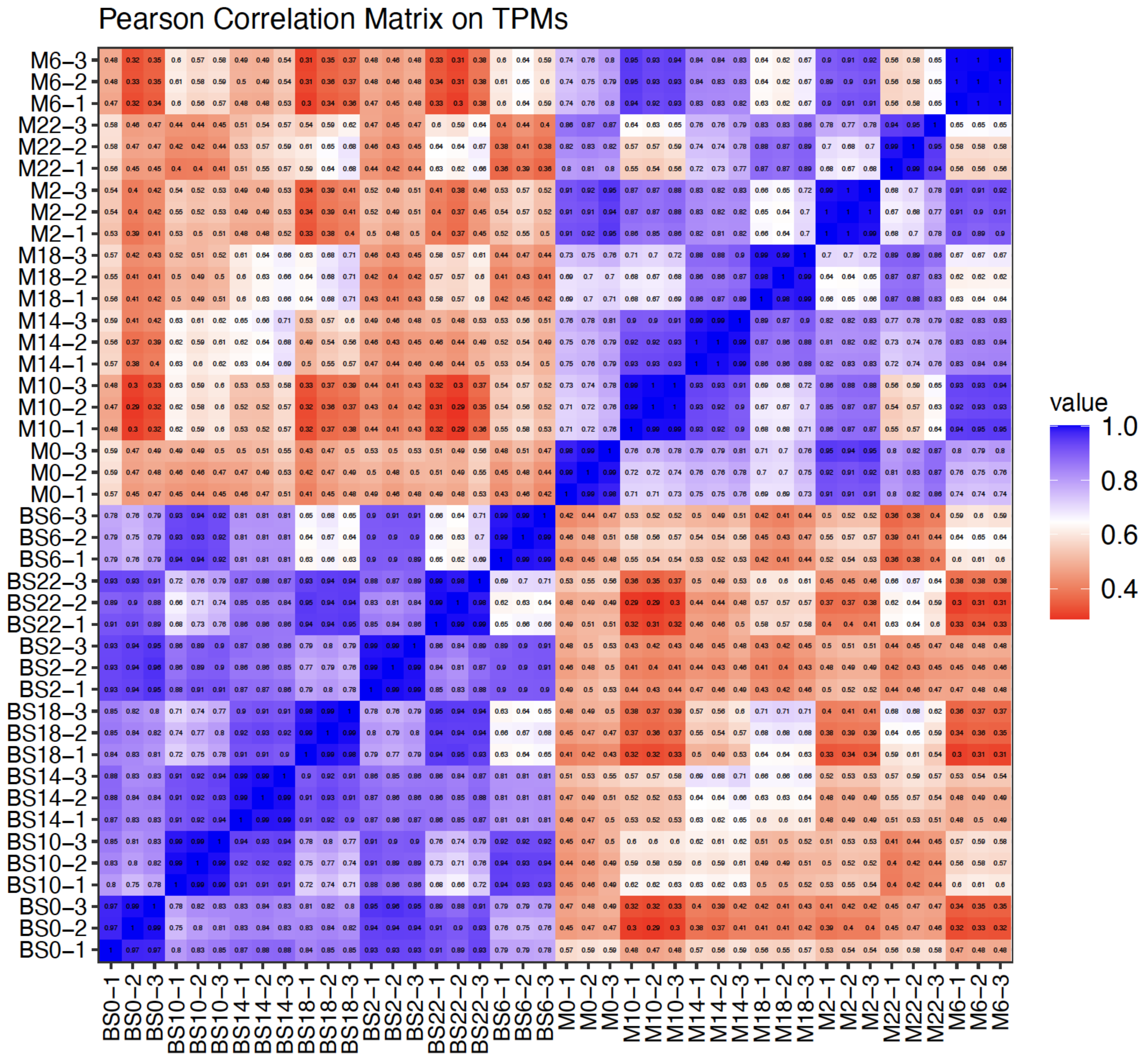

Supplemental Figure 1. Heatmap of Pearson's correlation coefficient (PCC) calculated between transcriptomes of biological replicates 1, 2 and 3 of mesophyll (M) and bundle sheath (BS) samples collected at time-points 0, 6, 10, 14, 18 and 22 hrs. High to low values of Pearson's correlation coefficient are shown as blue to red.

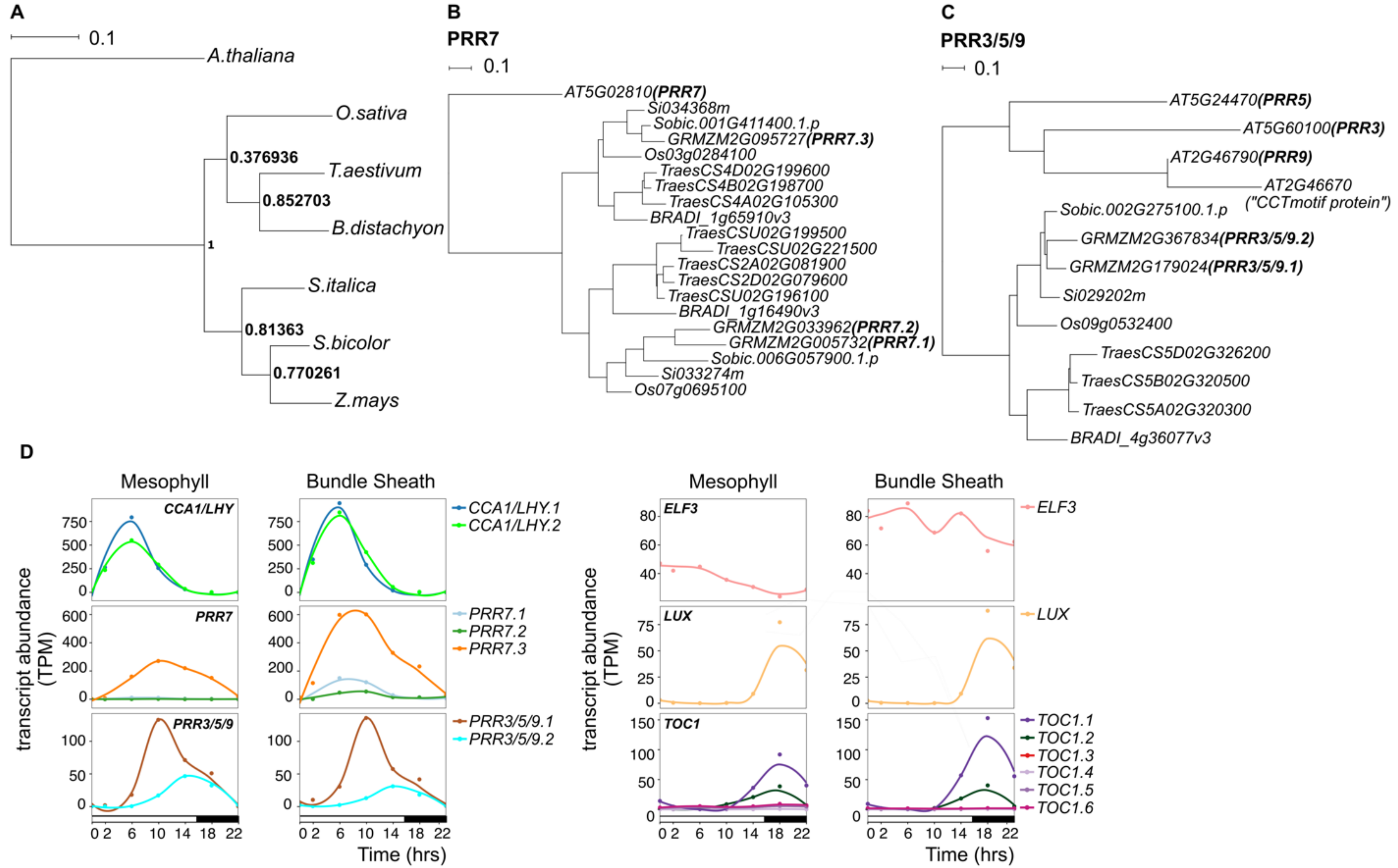

Supplemental Figure 2. Components of the maize circadian oscillator. **A)** Species tree inferred by *Orthofinder* with bootstrap values (100 bootstrap replicates) displayed at each node. **B-C)** Orthologue trees inferred for *PRR7* (B) and *PRR3/5/9* (C). The bars indicate evolutionary distance for each tree. **D)** Diel transcript abundance profile of genes for the circadian oscillator in mesophyll and bundle sheath cells. *x*-axis represents time-points and *y*-axis transcript abundance. TPM represents Transcripts Per Million reads. White and black bars on *x*-axis denote light and dark periods, respectively. *CCA1/LHY.1*, GRMZM2G014902; *CCA1/LHY.2*, GRMZM2G474769; *PRR7.1*, GRMZM2G005732; *PRR7.2*, GRMZM2G033962; *PRR7.3*, GRMZM2G095727; *PRR3/5/9.1*, GRMZM2G179024; *PRR3/5/9.2*, GRMZM2G367834; *ELF3*, GRMZM2G045275; *LUX*, GRMZM2G067702; *TOC1.1*, GRMZM2G148453; *TOC1.2*, GRMZM2G020081; *TOC1.3*, GRMZM2G066638; *TOC1.4*, GRMZM2G145058; *TOC1.5*, GRMZM2G174083; *TOC1.6*, GRMZM2G365688.

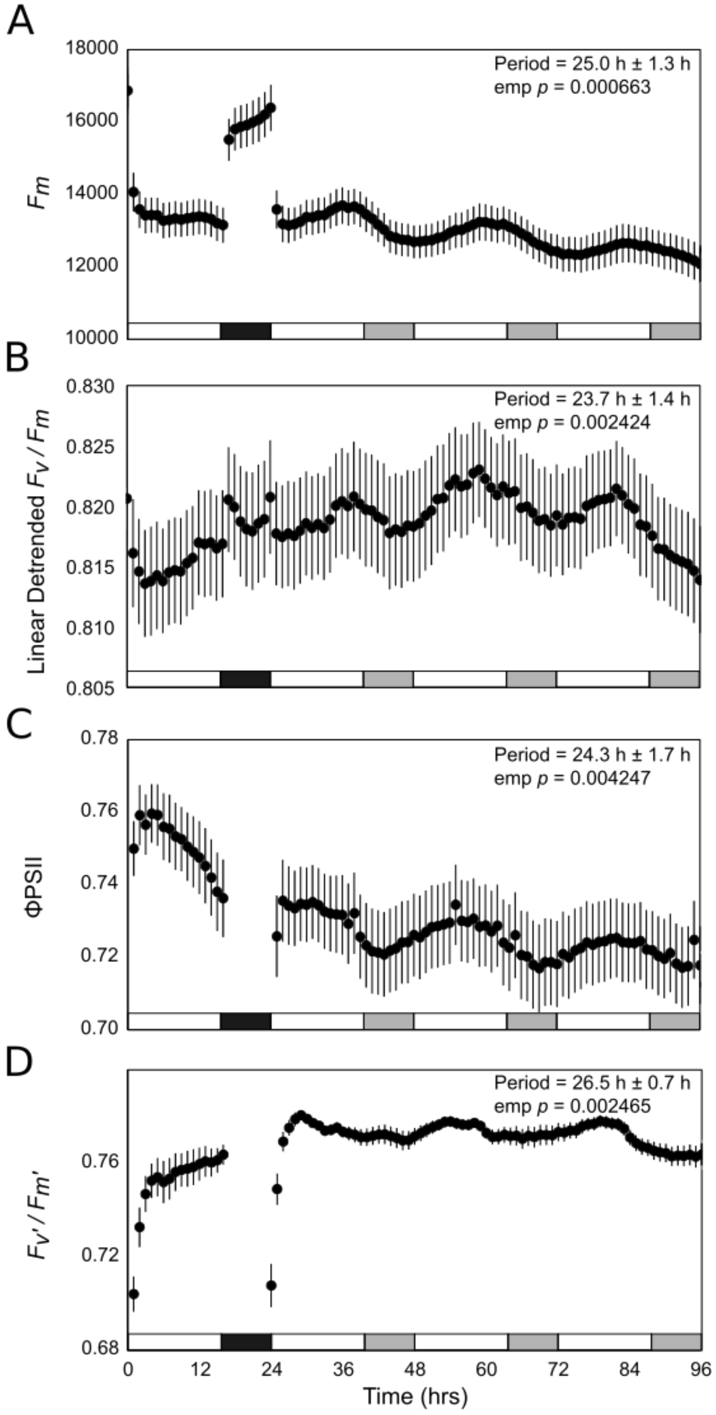

Supplemental Figure 3. Photosynthetic parameters measured for maize leaf fragments under one light-dark cycle (16 hrs light : 8 hrs dark) followed by 72 hours of a light regime that consisted of cycles of 40 minutes light and 20 minutes darkness. Data shown as mean with standard error (n = 6 biological replicates). **A)** F_m: maximum possible yield of fluorescence, **B)** Linear detrended F_v/F_m: maximum quantum efficiency of Photosystem II (PSII) photochemistry, **C)** ØPSII: operating efficiency of PSII, and D) F_v'/F_m': maximum efficiency of PSII photochemistry in the light. Black and grey bars represent dark period and subjective night, respectively. Empirical p-values calculated using the meta.meta function in MetaCycle from timepoints 48-96 hours in repeating light where emp $p < 0.01$ is considered rhythmic.

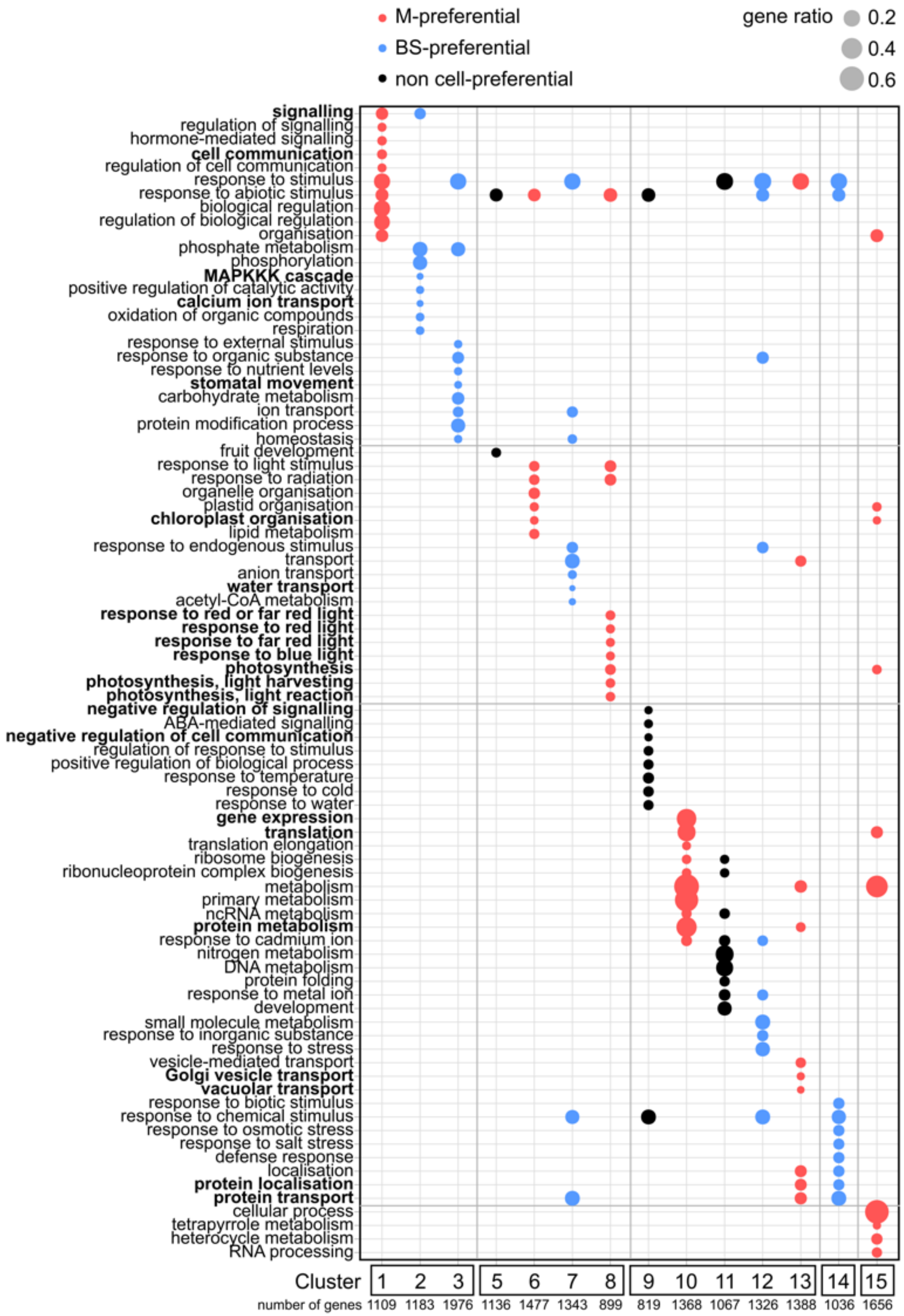

Supplemental Figure 4. Distribution of biological processes across the diel time-course and between mesophyll and bundle sheath cells. Dot plot showing the categories of biological processes with highest significance for each cluster (FDR $\leq$ 0.01). Clusters 1 to 3 peaked from dawn to 2 hours of light, clusters 5 to 8 from 6 to 10 hrs, clusters 9 to 13 from 14 to 22 hrs, cluster 14 at dawn and 22 hrs, and cluster 15 from dawn to 22 hrs. Gene ratio represents the proportion of genes assigned to a functional category in a cluster. M and BS represent mesophyll and bundle sheath cells, respectively.

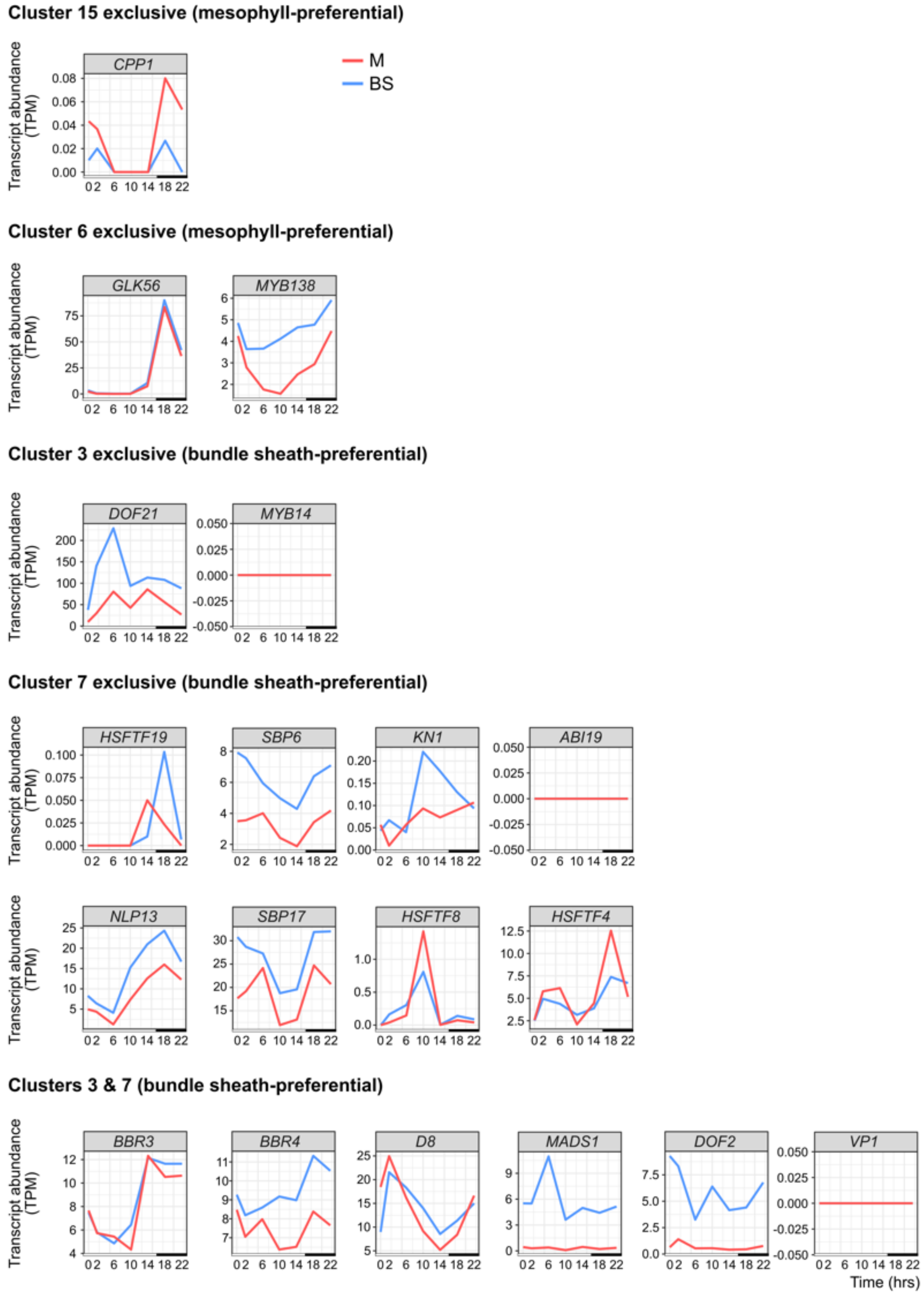

Supplemental Figure 5. Line plots representing the diel transcript abundance profile of genes encoding the cognate transcription factors for DNA-binding motifs enriched in mesophyll- and bundle sheath-preferential clusters of co-expressed genes. The *x*-axis represents time-points and the *y*-axis TPM values. TPM represents Transcripts Per Million reads. White and black bars in the *x*-axis denote light and dark periods, respectively. M and BS represent mesophyll and bundle sheath cells, respectively. CPP-transcription factor 1 (CPP1), GRMZM2G153754; G2-like-transcription factor 56 (GLK56), GRMZM2G067702; MYB-transcription factor 138 (MYB138), GRMZM2G139688; DNA-binding One Zinc Finger 21 (DOF21), GRMZM2G162749; MYB-transcription factor 14 (MYB14), GRMZM2G172327; HSF-transcription factor 19 (HSFTF19), AC216247.3_FG001; SBP-transcription factor 6 (SBP6), GRMZM2G138421; KNOTTED 1 (KN1), GRMZM2G017087; ABI3-VP1-transcription factor 19 (ABI19), GRMZM2G035701; NLP-transcription factor 13 (NLP13), GRMZM2G053298; SBP-transcription factor 17 (SBP17), GRMZM2G156756; HSF-transcription factor 8 (HSFTF8), GRMZM2G164909; HSF-transcription factor 4 (HSFTF4), GRMZM2G125969; BBR/BCP-transcription factor 3 (BBR3), GRMZM2G164735; BBR/BCP-transcription factor 4 (BBR4), GRMZM2G118690; Dwarf Plant 8 (D8), GRMZM2G144744; MADS-domain protein 1 (MADS1), GRMZM2G171365; DNA-binding One Zinc Finger 2 (DOF2), GRMZM2G009406; Viviparous 1 (VP1), GRMZM2G133398.

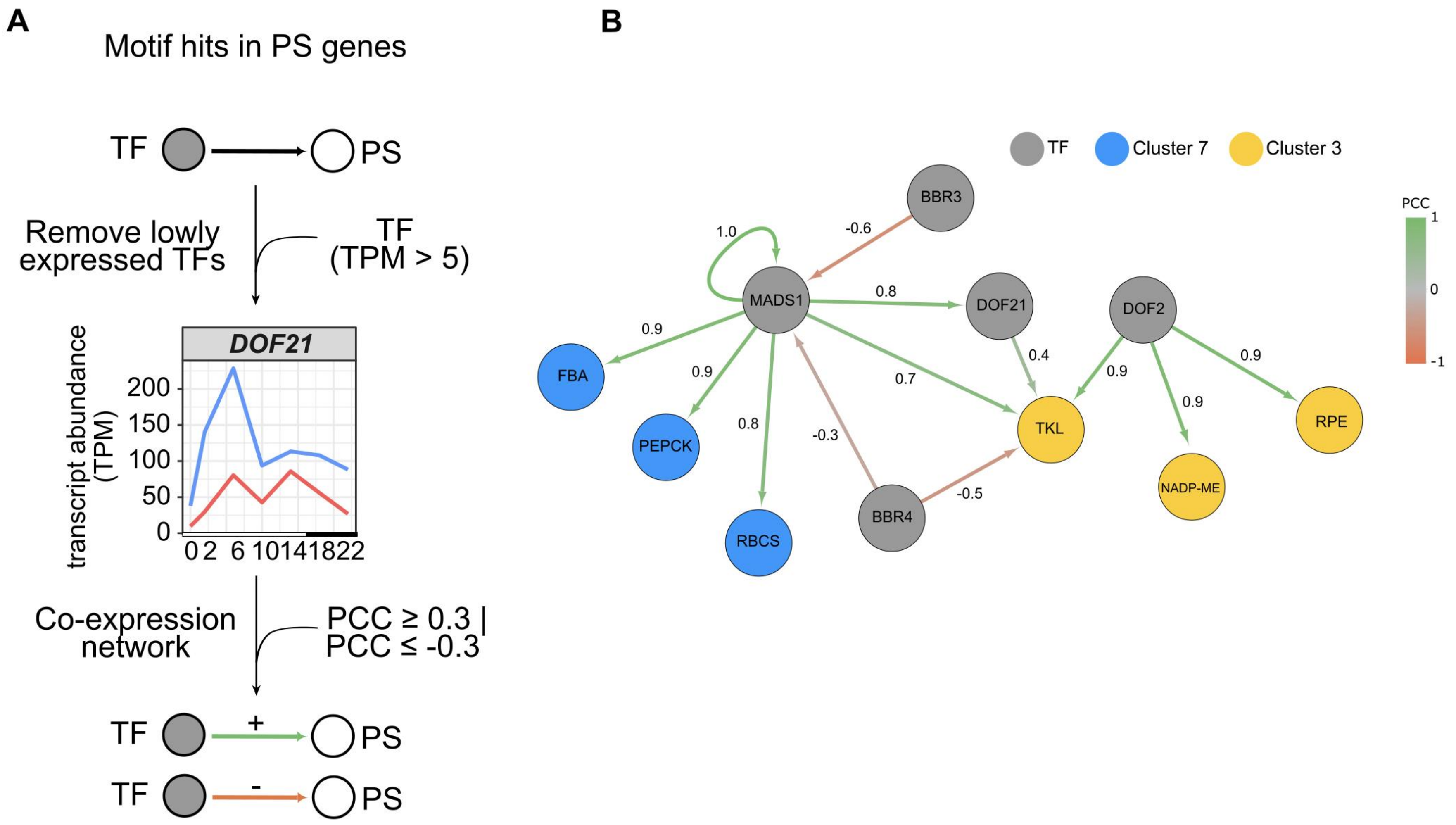

Supplemental Figure 6. Gene co-expression network built from RNA-seq data and DNA motif enrichment analysis. **A)** Transcripts encoding transcription factors (TF) with DNA-binding motif hits in photosynthesis (PS) genes (C_4 genes and Calvin-Benson-Bassham cycle genes) were filtered by their expression levels [Transcripts Per Million reads (TPM) > 5] and a gene co-expression network built for TF and PS genes using Pearson's correlation coefficient (cutoffs of < 0.3 and > -0.3). **B)** Gene co-expression network for TF and bundle sheath-preferential PS genes in clusters 7 and 3. Nodes represent TF (grey) and PS genes present in clusters 7 (dark blue) and 3 (yellow). Edges represent positive (green) and negative (red) co-expression based on the Pearson's correlation coefficient (PCC).
